# Avoidant Coping Style to High Imminence Threat Is Linked to Higher Anxiety-Like Behavior

**DOI:** 10.3389/fnbeh.2020.00034

**Published:** 2020-03-10

**Authors:** Shaun K. L. Quah, Gemma J. Cockcroft, Lauren McIver, Andrea M. Santangelo, Angela C. Roberts

**Affiliations:** ^1^Department of Physiology, Development and Neuroscience, University of Cambridge, Cambridge, United Kingdom; ^2^Behavioral and Clinical Neuroscience Institute, University of Cambridge, Cambridge, United Kingdom

**Keywords:** coping, anxiety, fear, threat, stress, emotion

## Abstract

Human studies with self-reported measures have suggested a link between an avoidant coping style and high anxiety. Here, using the common marmoset as a model, we characterize the latent factors underlying behavioral responses of these monkeys towards low and high imminence threat and investigate if a predominantly avoidant behavioral response to high imminence threat is associated with greater anxiety-like behavior in a context of low imminence threat. Exploratory factor analysis (EFA) of the human intruder test of low imminence threat revealed a single factor in which a combination of active vigilance and avoidance responses underpinned anxiety-like behavior. In contrast, two negatively-associated factors were revealed in the model snake test reflecting active and avoidant coping to high imminence threat. Subsequent analysis showed that animals with a predominantly avoidant coping style on the model snake test displayed higher anxiety-like behavior on the human intruder test, findings consistent with those described in humans. Together they illustrate the richness of the behavioral repertoire displayed by marmosets in low and high imminence threatening contexts and the additional insight that factor analysis can provide by identifying the latent factors underlying these complex behavioral datasets. They also highlight the translational value of this approach when studying the neural circuits underlying complex anxiety-like states in this primate model.

## Introduction

Anxiety and fear are key components of human emotion and have been described in the NIMH’s research domain criteria as adaptive responses to potential threat and acute threat respectively. Anxiety and fear may also be differentiated based on their position on the predatory-threat imminence continuum; with imminence being influenced by temporal, spatial, and probabilistic closeness to the threat, as well as other threat characteristics (Perusini and Fanselow, 2015). Anxiety is hypothesized to drive pre-encounter defensive behaviors when imminence is considered to be low and there is a high level of uncertainty or ambiguity, e.g., increased vigilance for risk assessment (Blanchard et al., 2011). In contrast, fear is hypothesized to drive post-encounter defense behaviors when imminence is considered to be high, e.g., freezing, attack.

Distortion of threat imminence and dysregulated defensive behaviors may form the core symptomatology of anxiety disorders. Individuals with high trait anxiety, a natural disposition to attend to, experience and report negative emotions across many situations, have increased risk of developing anxiety disorders and depression (Weger and Sandi, 2018), and display greater responsivity to threat cues (Indovina et al., 2011). Maladaptive coping patterns to threatening stimuli may play a role in the dysfunctional regulation of emotion observed in patients with anxiety disorders. Specifically, studies of self-report measures in humans have suggested that the tendency to adopt an avoidant coping strategy is linked to anxiety and depressive symptoms during adolescence (Chan, 1995; Herman-Stabl et al., 1995; Seiffge-Krenke and Klessinger, 2000; Gomez and McLaren, 2006), and increased post-trauma PTSD symptom severity (Pineles et al., 2011).

The human literature of coping inventories and questionnaires broadly delineates coping into active and avoidant strategies (Herman-Stabl et al., 1995; Seiffge-Krenke and Klessinger, 2000; Frydenberg and Lewis, 2009; Pineles et al., 2011). Active and avoidant coping refers to cognitive or behavioral activity either towards (active) or away (avoidant) from the threat, sometimes simplified as fight-or-flight. Similarly, animal studies of coping in highly stressful situations categorize responding to the active and avoidant dimensions (Koolhaas et al., 1999). For example, “active/proactive” rats display more aggressive behavior in response to an intruder and spend more time actively burying shock probes, whereas “avoidant/reactive” rats display less aggressive behaviors to an intruder and spend more time being immobile in the defensive burying test (Koolhaas et al., 2010).

To study these complex latent constructs representative of human anxiety and fear in an animal model, first, we should determine whether a similar relationship between coping styles and anxiety is reflected in animals. This requires an approach that can properly represent the construct driving the diverse repertoire of behaviors that animals display across different situations involving low and high imminence threat. Unfortunately, preclinical paradigms often rely on simple unidimensional measures of anxiety-like and fear-like animal behavior that only bear a weak resemblance to human anxious and fear-driven behavior. While these measures may have predictive validity, e.g., rodent’s tendency to stay in enclosed spaces in an anxiety-provoking context is sensitive to anxiolytics (Borsini et al., 2002), the use of these measures to represent latent constructs may be an oversimplification. Behaviors such as an animal’s tendency to stay in enclosed spaces are likely driven by multiple underlying factors such as an animal’s territoriality or propensity for exploration, and not just anxiety *per se*. Indeed, misattributing these observed effects may contribute to the current difficulty in translating findings from animal studies to humans. Thus, a multivariate approach modeling the underlying latent construct driving the observed behaviors is needed.

Such an approach has been used in macaques (Williamson et al., 2003; Fox et al., 2008) and marmosets (Agustín-Pavón et al., 2012; Shiba et al., 2014) in tests measuring responsivity when there is low (human intruder test) and high (model snake test) imminence threat with composite or principal component scores. However, a limitation of using these simple composite scores is that it only simplifies the data and does not determine the latent variables driving the observed changes within the data. Instead, factor analysis is widely utilized in validation studies of psychological tests and has recently been used to uncover the latent variables affecting the behavioral response of rhesus macaques in the human intruder test (Gottlieb and Capitanio, 2013).

Thus, in the present study, we applied an exploratory factor analysis (EFA) to characterize the factors underlying the common marmoset’s behavior in response to both low threat imminence (the human intruder test) and high threat imminence (the model snake test) in order to reveal the relationship between coping styles in the high threat imminence context and anxiety-like behavior (in a low threat imminence context) as reported in self-report studies in human.

## Materials and Methods

### Subjects

All animals were bred on-site at the Innes Marmoset Colony (Behavioral and Clinical Neuroscience Institute, BCNI) and when adult, pair-housed predominantly (~90%) as unrelated male-female pairs (males were vasectomized). Temperature (22 ± 1°C) and humidity (50 ± 1%) conditions were controlled and a dawn/dusk-like 12 h-period was maintained. They were provided with a balanced diet and water *ad libitum*. All procedures were performed in accordance with the project and personal licenses held by the authors under the UK Animals (Scientific Procedures) Act 1986.

The total tested population consists of 184 common marmosets (*Callithrix jacchus*). 171 animals (sex (M/F) = 90/81; age: 2.32 ± 0.62 years) were tested with the human intruder test and 151 common marmosets (sex (M/F) = 77/74; age: 2.51 ± 0.68 years) were tested with the model snake test. Of these, 134 (sex (M/F) = 71/63) were tested on both the human intruder test (age: 2.29 ± 0.62 years) and model snake test (age: 2.5 ± 0.68). For animals tested on both tests, the human intruder test was conducted before the model snake test (time between test (months): 2.5 ± 4). Although the order of these tests was not counterbalanced, these two tests used completely different stimulus types and were conducted at least 3 days apart, thus, whilst a potential effect of test order cannot be ruled out, it is unlikely.

All animals received either the human intruder and/or model snake test in early adulthood after having left the family group and been paired with a cagemate of similar age for at least 1 month. The majority of these animals (139 out of 184) formed part of the screening procedure within the colony allowing an animal’s emotional reactivity to be assessed prior to entering an experimental protocol. Only thirty-nine of these were not completely naïve, having received anesthesia for restraint purposes only while undergoing MRI scanning during development and early adulthood. The remaining animals (45) were tested on the human intruder and snake tests prior to the introduction of the screening procedure and had received additional non-related procedures beforehand. Twenty-nine had received a telemetry probe into the descending aorta, and sixteen received a single dose of 5-HT_2A_ receptor radioligand (altanserin) as part of PET scanning. No animal received the human intruder or model snake test within a week of these procedures.

### Testing Apparatus

Both tests were performed in the top right-hand quadrant of the animal’s home-cage [92 cm (high) × 60 cm (wide) × 98 cm and 73 cm (length of sides)] and were conducted in the presence of conspecifics in adjacent cages. A typical home cage room contains approximately 22–28 animals. Under these conditions, the subject displays a richer repertoire of behaviors in an aversive or ambiguous context than would be seen if the animal was fully isolated. Indeed, unpublished observations from our own laboratory show us that marmosets make little in the way of vocalizations or display active coping behaviors when confronted with an unknown human in isolation and individual differences are less marked. As it is not possible to control for the behavior of neighboring animals in the room, it cannot be ruled out that they may influence the behavior of the subject. But given that up to 184 animals were tested across nine different home cage rooms, it is unlikely that any specific effect of conspecifics on any individual had a significant effect on the overall dataset.

### Human Intruder Test

The human intruder test involves measuring the animal’s behavioral response to an unfamiliar human, the “human intruder,” who stands in front of the animal’s home-cage and maintains eye contact with the animal. Since animals bred in the laboratory have prior positive and negative experiences with human encounters, e.g., receiving food treats or being restrained for husbandry or experimental purposes, the unfamiliar “human intruder” acts as a threat with low probabilistic imminence and creates an anxiety-provoking context. Avoidance and vigilance during the task resemble that described for human anxious behavior (reviewed in Grupe and Nitschke, 2013), and behavioral responses to the human intruder are sensitive to anxiolytics (Carey et al., 1992; Santangelo et al., 2016).

The procedure for the human intruder test is based on the method used by Santangelo et al. (2016). Cameras and microphones are routinely present in the room for recording purposes such that all animals are habituated to the presence of recording equipment. Before the testing session begins, a camera and microphone are set up in front of the animal’s home-cage. The animal was tested in the top-right quadrant of their home cage (Supplementary Figure S1). During testing, the cagemate was separated from the subject and restricted to the left half of the home cage and was obscured from both the human intruder and the subject. After 8 min of being separated, an experimenter (unfamiliar to the animal) wearing a realistic latex human mask (Greyland Film, UK) and standard lab attire stood 40 cm from the cage and maintained eye contact with the subject for 2 min (intruder phase). Recording continued for a further 5 min after the intruder left (recovery phase). Behavior and vocalizations during the intruder phase were scored.

**Figure 1:**
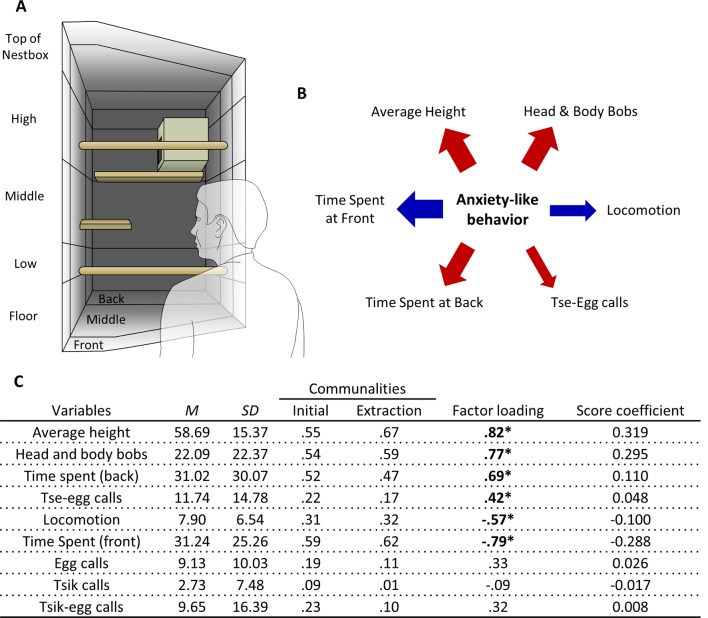
Human Intruder test exploratory factor analysis (EFA). **(A)** Schematic of the top-right quadrant of the home-cage in which the human intruder test takes place, with relevant zones for the measurement of average height, and time spent at the front and back. **(B)** The relative contribution of each behavioral measure loading significantly on the factor representing anxiety-like behavior reflected by the width of the arrow. Red arrows represent positive-loading; blue arrows signify negative-loading. Positive loadings indicate that higher anxiety-like behavior corresponds to an increase in that specific measure, while a negative loading indicates a decrease. **(C)** Table of descriptive statistics, communalities, factor loadings and factor score coefficients for the variables in the human intruder test. *Significant factor loadings (>|0.4|) in bold. Mean (*M*) and standard deviation (SD) of variables from the cohort (*n* = 171).

**Figure 2:**
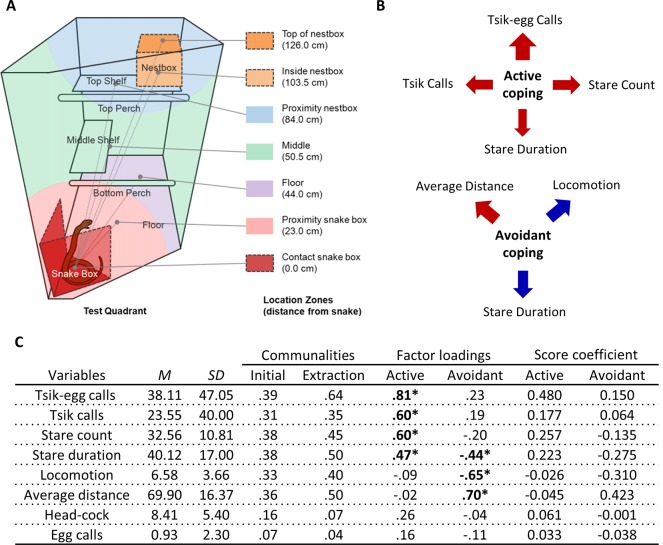
Rubber snake test EFA. **(A)** Schematic from Shiba (2013) of the top-right quadrant of the home-cage with the snake box in the rubber snake test. Zones are depicted in different colors indicating the mean distances those zones represent relative to the rubber snake model. Similar to the human intruder test, the cagemate is separated into the left half of the homecage by dividers (not shown). **(B)** The relative contribution of each behavioral measure in the rubber snake test to the coping factor scores reflected by the width of the arrow. Red arrows represent positive-loading; blue arrows signify negative-loading. Positive loadings indicate that higher coping scores correspond to an increase in that specific measure, while a negative loading indicates a decrease. **(C)** Table of descriptive statistics, communalities, factor loadings and factor score coefficients for the variables in the rubber snake test. *Significant factor loadings (>|0.4|) in bold. Mean (*M*) and SD of variables from the cohort (*n* = 151).

The animal’s observable behavior was scored using the program JWatcher V1.0^1^. For the purposes of scoring, the test quadrant was divided into multiple zones represented by different depths and heights (Figures 1A, 2A). While, as described below, the average distance from the threat was used in the model snake test, this measure was not used in the human intruder test because the position of the threat in the model snake test can be reduced to a single point relative to the different positioning of the animal. In contrast, in the human intruder test, the “human intruder” facing the animal covers a larger area and the animal’s position relative to the “human intruder” is better represented independently by depth and height instead. Furthermore, both time spent at the front of the cage and the back of the cage were used as measures of approach-avoidance behavior due to studies showing the sensitivity of these measures to anxiolytic and anxiogenic manipulations (Carey et al., 1992).

### Human Intruder Test: Behavioral Measures

#### Time Spent at the Front (TSAF)

Percentage time spent at the front of the cage reflects approach behavior towards the human intruder. For the purposes of scoring, the test quadrant was divided into 3 zones: front, middle, and back. These different zones represent the depth of the zone relative to the “human intruder.”

#### Time Spent at the Back

Percentage of time spent at the back of the cage reflects avoidance behavior away from the human intruder. Scored similarly to “Time spent at the front.”

#### Average Height

Average height of the marmoset in the home-cage throughout the test period in centimeters. Positioning high in the cage and closer to the nestbox may reflect the common marmoset’s innate flight response upwards as an arboreal species. For scoring purposes, the test quadrant is divided into five different zones: top of the nestbox, high, middle, low, floor. These different zones represent the height of the zones relative to the bottom of the test quadrant (shown in Figure 1).

#### Locomotion

Percentage of time spent changing locations within the home-cage.

#### Head and Body Bob

Frequency of the animal making rapid bobs of its head and body from side to side (without changing head angle) while staring at the object of interest and is often followed with egg and tse-egg vocalizations (see descriptions below). Head and body bobs are often observed in the presence of an unfamiliar human and may be an alarm behavior intended to signal potential threats to conspecifics (Carey et al., 1992; Agustín-Pavón et al., 2012).

### Model Snake Test

The model snake test involves recording the animal’s behavioral response to a rubber snake which acts as an inherent predatory stimulus, provoking an innate fear response (Barros et al., 2002; Cross and Rogers, 2006). Furthermore, as the model snake is placed directly within the homecage, the model snake presents far higher spatial threat imminence compared to the intruder in the human intruder test.

The procedure of the model snake test is based on the methods in Shiba et al. (2014). Before the testing session begins, wireless cameras and a microphone are placed to record the animal’s behavior from a top-down view and a frontal view. During a test session, the animal is separated from their cagemate and restricted to the upper right quadrant of their home cage (Supplementary Figure S1), while the cagemate was separated by opaque dividers to the left half of the home cage and cannot see into the testing quadrant. The 20-min test session is divided into four 5-min phases: a separation phase, where only the camera and microphone were present; a pre-snake phase, where an empty box without the model snake (a 27 cm tall rubber model of a rearing cobra) is placed in the test quadrant; a snake phase, where the empty box from the previous phase is replaced with a box containing the model snake (a sliding door is removed to expose the model snake once the box is in position); and a post-snake phase, where the empty box from the pre-snake phase is re-introduced into the test quadrant.

### Model Snake Test: Behavioral Measures

#### Average Distance

Average distance of the marmoset from the model snake throughout the test period. For scoring purposes, the test area was divided into seven zones based on their proximity to the model snake (shown in Figure 2). Each zone is represented by the distance of the mid-point of that zone from the snake. The average distance is calculated by obtaining the sum of the multiplication of the percentage time spent in each zone with the distance of the respective zones.

#### Locomotion

Percentage of time spent changing locations around the home-cage.

#### Stare Duration

Percentage time the animal spent maintaining eye and head orientation directed towards the model snake.

#### Stare Count

Number of times the animal spends directing its attention towards the snake. Multiple counts indicate looking away and back towards the model snake and reflect an animal repeatedly averting its gaze away from the snake but clearly pre-occupied with the snake.

#### Head-Cock

Frequency of the animal tilting its head in a smooth motion while maintaining its attention towards the visual target. Head-cocks have been described as an observational behavior when presented with a novel stimulus and occur during visual inspection (Menzel, 1980; Barros et al., 2002).

### Vocalizations in the Human Intruder and Model Snake Tests

The animal’s vocalizations recorded with a directional microphone to isolate vocalizations from the subject were extracted from the video files using Audacity, an audio editing software (Audacity, v.1.3.13) and subsequently visualized in the form of sonograms using Syrinx, a sound analysis software. The scoring of vocalizations was guided by the video recording to confirm the subject as the source of the vocalizations. Classification of vocalizations was based on identifications from Bezerra and Souto (2008) observation of wild common marmosets. Although other calls such as phee, twitter, and bark were observed, they occurred very infrequently and only in a small subset of the population which lead to their exclusion from this study.

#### Egg Calls

A short call with a few harmonics. Maybe uttered singly, in series, or in continuous combination after tse or tsik calls. Egg calls have been associated with vigilance behavior, for instance when an unknown human approaches the group or when the calling marmoset is on the ground with sparse vegetation (Souto et al., 2007). Primarily heard in response to human intruder and seldom heard in response to snake.

#### Tsik Calls

Tsik calls are uttered as a mobbing call and have been observed being made by captive and wild common marmosets against conspecifics from other social groups, unfamiliar humans, and potential predators (Epple, 1968; Bezerra et al., 2009). Tsik calls have also been observed being made by captive common marmosets in response to the stimulus presentation of a predator (Hook-Costigan and Rogers, 1998; Cross and Rogers, 2006).

#### Tsik-Egg Calls

Although not clearly characterized in the wild, tsik-egg calls of common marmosets have been associated with isolation in a novel environment and have been shown to be sensitive to anxiogenic drug treatment (Kato et al., 2014).

#### Tse Calls

Sounds similar to tsik calls but distinguishable *via* sonogram. The lower frequency and end frequency of tse calls are higher than tsik calls. The frequency range in tse calls is also lower than tsik calls (Bezerra and Souto, 2008).

#### Tse-Egg Calls

A vocalization consisting of a single utterance of tse followed by a single or a series of egg calls. In the wild, tse-egg calls are the primary call type uttered during vigilance behavior (89.2% and 80.4% of total calls during vigilance in adults and juveniles respectively; Bezerra and Souto, 2008).

### Exploratory Factor Analysis (EFA)

All statistical analyses were conducted with SPSS (v. 24; IBM Corp.). An EFA with a principal axis factoring extraction method was performed on the data obtained from the human intruder and model snake tests separately. The principal axis factoring extraction method was used as the variables revealed violations of normal distribution (shown in Supplementary Figures S2, S3) and principal axis factoring does not assume a multivariate normal distribution.

#### Pre-factor Extraction Tests

Before factor extraction, the Kaiser-Meyer-Olkin measure of sampling adequacy (MSA) was used to determine the proportion of common variance among the variables that may be driven by underlying factors. The Bartlett’s test of sphericity was used to evaluate if there were sufficient correlations between the variables such that the factor analysis is able to model underlying constructs driving these correlations.

#### Post-factor Extraction

After factor extraction, the communality of a variable is the extent to which that variable correlates with all other variables in the analysis. If the average communality of the variables is more than 0.7 after extraction, the Kaiser’s criterion (eigenvalue > 1) should be used to determine the number of factors to extract, otherwise, the scree plot’s points of inflection should be referred to instead (Field, 2013). The scree plot shows the eigenvalue, which reflects the amount of variance explained, of each individual factor.

#### Rotation

If more than 1 factor is extracted, the factors are rotated to improve the interpretability of the resulting factors by maximizing the loadings of each variable to a specific factor and minimizing loading on other factors. A direct oblimin method (oblique rotation) was used to allow for correlations between the factors as there were no theoretical grounds to assume the independence of the factors.

After the factors were extracted and rotated, the factor loadings could be referred to as a measure of each variable’s correlation with the extracted factor. Factor loadings are considered significant above |0.4| (Stevens, 1992). To measure the goodness-of-fit for the extracted factor model, a correlation matrix was constructed based on the model and the difference (residuals) between the reproduced correlation matrix and the original correlation matrix computed. The proportion of nonredundant residuals with absolute values greater than 0.05 should be below the recommended value of 50% if the factor model does not have issues of poor fit (Field, 2013). The factor scores were estimated with a regression method, preserving any existing correlation between the factors.

The internal consistency of the factors with significant loading variables was examined using Cronbach’s alpha. Cronbach’s alpha evaluates how consistently the factor reflects the construct it is measuring (Cronbach, 1951).

### Correlation Between Model Snake Test Factor Scores

The resulting factor scores of the model snake test were correlated using Pearson’s product-moment correlation coefficient or, if the assumption of normality was severely violated (*p* < 0.001), using Spearman rank correlation coefficient. Data are presented as mean ± SEM, standard error of the mean. Effect sizes of correlations are reflected in the correlation coefficients, r_s_ (Cohen, 1988, 1992).

## Results

### EFA Reveals a Single Factor Reflecting Avoidance and Vigilance to Explain Behavior in Response to a Human Intruder

A single factor explained behavior on the human intruder test (Figure 1A). Those behaviors that contributed greatest to the factor were the time spent at the front and back of the cage, average height, and head and body bob. Locomotion and tse-egg calls also contributed (Figure 1B). Highest scores were associated with greater avoidance (more time spent at the back of the cage and relatively high up) and increased vigilance (making little movement, performing a greater number of head and body bobs and tse-egg calls).

To derive this factor, initial runs of the EFA included: time spent at the front, time spent at the back, average height, locomotion, head and body bobs, egg calls, tsik call, tsik-egg calls, tse calls, and tse-egg calls. The variable with the lowest MSA that was below the standard of 0.5 defined by Field (2013), tse calls (MSA = 0.42) was removed from the EFA. Subsequently, the KMO MSA for the final model indicated sufficient common variance for the factor analysis, KMO = 0.82, well above the recommended threshold of 0.6 (Kaiser, 1974). Bartlett’s test of sphericity was significant ($\chi_{\left(36\right)}^{2}$ = 460.8, *p* < 0.001), indicating that correlations between items were sufficiently large for factor analysis. Due to the low level of communalities, reflecting low inter-variable correlations, after extraction (Figure 1C), the scree plot was consulted to decide the number of factors to extract, instead of using Kaiser’s criterion. The factor coefficient matrix estimated from the final output of the EFA and descriptive statistics of the sample is also shown in Figure 1C. Only 1 factor was extracted based on the point of inflection on the scree plot (Supplementary Figure S4A). This factor accounted for 39.7% of the total variance. There were 16 (44.0%) nonredundant residuals, reflecting the sufficient fit of the one-factor model.

The factor (as described above) with 6 significant loading items had moderate reliability, Cronbach’s alpha = 0.64. Kline (2000) notes that psychological constructs with Cronbach’s alpha below 0.7 should be realistically expected. Eliminating individual variables from the factor did not yield substantial increases to the alpha measure.

### EFA Reveals Two Negatively Correlated Factors Reflecting Active and Avoidant Coping to Explain Behaviors in Response to a Model Snake

Two factors described behaviors elicited on the model snake test (Figure 2A). The first factor included attention towards the snake (long durations spent staring at the model snake and higher frequencies of re-attending to the model snake after looking away) and mobbing calls (tsik-egg and tsik calls). Those scoring high on this factor displayed heightened attentional engagement and increased mobbing calls, altogether reflecting an active coping response. The second factor included distance, locomotion and stare duration. A high score was characterized by an animal maintaining a greater distance from the model snake, remaining relatively stationary and spending less time staring at the snake, reflecting overall behavioral and attentional avoidance of the snake (Figure 2B).

To derive these factors, initial runs of the EFA included: average distance, locomotion, stare duration, stare count, head-cocks, egg calls, tsik call, tsik-egg calls, tse calls, and tse-egg calls. Tse calls (MSA = 0.32), the variable with the lowest MSA that was below the criterion of 0.5 defined by Field (2013), were removed from the EFA. Under the same criterion (MSA = 0.46), tse-egg calls were removed in the subsequent run. The KMO MSA for the final model indicated sufficient common variance for the factor analysis, KMO = 0.63, just above the recommended threshold of 0.6 (Kaiser, 1974). Bartlett’s test of sphericity was significant ($\chi_{\left(28\right)}^{2}$ = 233.1, *p* < 0.001), indicating that correlations between items were sufficiently large for factor analysis. Due to the low level of communalities after extraction (Figure 2C), the scree plot was consulted to decide the number of factors to extract instead of using Kaiser’s criterion (Field, 2013). The factor loadings, factor score coefficients estimated from the final output of the EFA and descriptive statistics of the sample are also shown in Figure 2C. Two factors were extracted based on the point of inflection on the scree plot (Supplementary Figure S4B) for 50.3% of the total variance. There were 10 (35.0%) nonredundant residuals, indicating that the two-factor model does not have issues of poor fit.

Factor 1, that reflected active coping behaviors, consisted of 4 significant loading items and had moderate reliability, Cronbach’s alpha = 0.61. Factor 2, that reflected avoidant coping behaviors, consisted of 3 significant loading items and had relatively lower reliability, Cronbach’s alpha = 0.52. This may be due, in part, to the low number of variables contributing to this factor.

**Figure 3:**
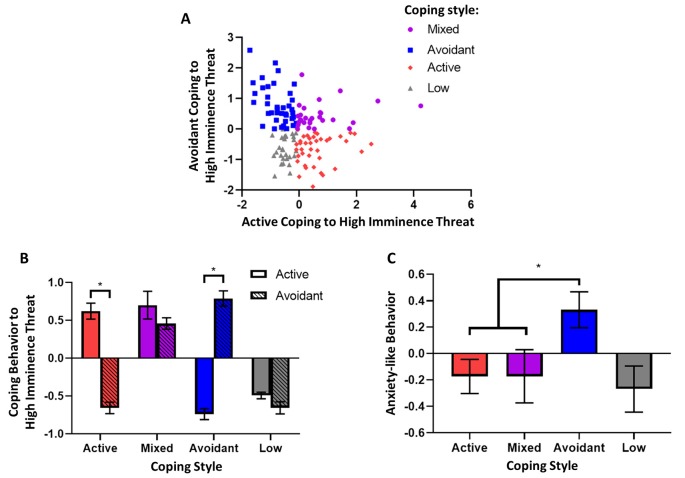
Coping style to high imminence threat and associated anxiety-like behavior. **(A)** Factors representing active and avoidant coping with high imminence threat in the rubber snake test were significantly negatively correlated (Spearman’s, *p* < 0.005). **(B)** Animals were grouped based on their coping style to high imminence threat: animals grouped as having active coping styles had significantly higher active coping factor scores compared to avoidant coping scores, while the opposite was true for animals grouped as having avoidant coping styles. For animals grouped as having a mixed coping style, coping scores were both above the mean and not significantly different. Lastly, animals that had low responsivity in the test had coping scores that were both below the mean and not significantly different. **(C)** Animals with an avoidant coping style had higher levels of anxiety-like behavior as measured by the human intruder test factor score. *p < 0.05. Error bars represent SEM.

A comparison of the two factors showed that they were negatively correlated with one another. Active coping behavior was significantly negatively correlated (nonlinear) with avoidant coping behavior (*r_s_* = −0.25, *p* = 0.002) with a small to medium effect size (0.1 < |*r|* < 0.3; Figure 3A), indicating that behaviors corresponding to actively attending to the model snake are negatively associated with avoidant behaviors towards the model snake. The nonparametric Spearman’s rank-order correlation was used to determine the relationship between the factors as the factor representing active coping severely violated the assumption of normality (*W*_(151)_ = 0.93, *p* < 0.001, Supplementary Figure S5).

### Animals Who Display High Avoidance and Low Active Coping to High Imminence Threat Show the Highest Levels of Anxiety-Like Behavior to Low Imminence Threat

Finally, it was determined whether animals with a predominantly avoidant coping style in response to the high imminence threat of the model snake, display higher levels of anxiety-like behavior to the low imminence threat of the human intruder. Accordingly, animals receiving both tests were grouped according to their overall response style to the snake (Figure 3B). First, active and avoidant coping factor scores from the model snake test were categorized as high if they were above the median of the population and low if they were below the median (advantages and disadvantages of a median split discussed in Allen, 2017). Subsequently, animals with high avoidant but low active coping scores were grouped as animals with an avoidant coping style (*n* = 39); animals with low avoidant but high active coping scores were grouped as having an active avoidant coping style (*n* = 39), and animals with both high avoidant and high active coping scores were grouped as animals with mixed coping styles (*n* = 28). Animals that showed overall low reactivity towards the model snake (both low avoidant and low active coping scores, *n* = 28) did not show a specific coping style and were not included in the subsequent group comparison. The group’s distinct distribution of factor scores is shown in Figure 3B.

A subsequent analysis comparing these groups’ respect to their corresponding behavior towards a human intruder revealed that animals with an avoidant coping style showed greater anxiety-like behavior to the human intruder in comparison to all other groups. Specifically, there was a significant effect of coping style (*F*_(2,103)_ = 3.91, *p* = 0.023) on anxiety-like avoidant/vigilant behavior, and a Dunnett’s *post hoc* comparison revealed that animals with a selectively avoidant coping style had higher anxiety-like behavior compared to both animals with a selectively active coping style (*p* = 0.027) and those with a mixed coping style (*p* = 0.048; Figure 3C).

## Discussion

Although preclinical research with animal models has made substantial contributions to our understanding of emotion regulation in response to the threat, the behavioral substrates underlying these latent constructs remain simply represented and the relationship between these constructs remains poorly understood. Here, in a nonhuman primate, we modeled the factors driving behavior in the context of low imminence (human intruder test) and high imminence (model snake test) threat and established a relationship between these underlying constructs.

**Figure 4:**
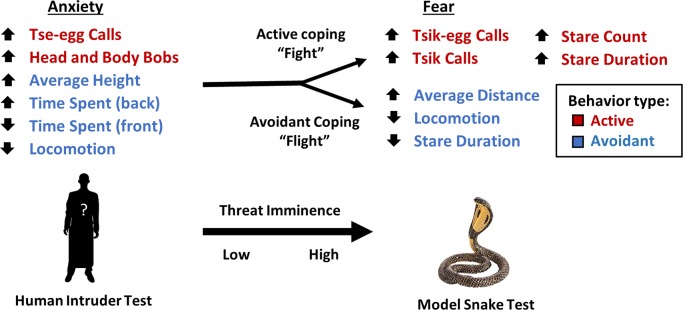
Transition of behaviors in response to low and high imminence threat. As an animal’s appraisal of threat transitions from low to high imminence, an animal’s behavioral pattern shifts from a combination of both active (red) and avoidant behaviors (blue) to either a “fight” response characterized by active behaviors to confront the threat or a “flight” response characterized by attempts to avoid confrontation with the threat. The direction of arrows for variables indicates the direction of factor loading.

In relation to an unknown human, EFA of data from 171 marmosets yielded one underlying factor driving the observed behaviors. We interpret this factor as reflecting the animal’s anxious temperament as it includes behavior typically associated with high levels of anxiety-like responses. Specifically, an animal with a high anxiety factor score is characterized by marked avoidance behavior that includes spending more time at positions further away from the human intruder (higher up and at the back of the cage), less time at positions close to the human intruder (the front of the cage) and less time moving around (low locomotion). Moreover, they display marked vigilance behavior including head and body bobs and vigilance calls (tse-egg calls). This pattern is similar to that shown by marmosets in the wild, which predominantly make tse-egg calls when being vigilant of their surroundings, peer into the vegetation while being stationary and make head and body bobs (Bezerra and Souto, 2008). The uncertainty and anticipation model of anxiety posits that behavioral and cognitive avoidance and increased threat vigilance are among the key psychological processes central to the increased threat expectancies of subclinical and clinical anxiety (Grupe and Nitschke, 2013). Taken together, the putative anxiety factor in the human intruder test reflects classic components of anxiety-like behavior, specifically avoidance and active vigilance (Figure 4).

In contrast, the EFA of data from 151 animals confronted by the model snake yielded two underlying factors driving the observed behaviors. The two factors may be interpreted as reflecting the animal’s active and avoidant coping response. A higher active coping response (factor 1) includes higher frequencies of mobbing calls (tsik-egg and tsik) in the presence of the predatory stimulus as well as increased attention towards the predatory stimulus, namely longer durations of staring at the model snake and higher levels of re-diverting attention towards the snake (measured by stare count). The mobbing calls serve to alert conspecifics of a potential predator and to drive predators away (Epple, 1968). Tsik calls, in particular, have also been associated with reduced cortisol levels, implicating mobbing behavior in the reduction of physiological stress (Cross and Rogers, 2006). Thus, overall, this active coping factor consists of attentional engagement and vocalizations that may underlie the animal’s attempt to confront and overcome the threat (Figure 4). In contrast, a higher avoidant coping response (factor 2), including higher average distance, lower locomotion, and lower stare duration serve to avoid contact between the animal and the threat (model snake) and avoid drawing attention to the animal.

While these two factors in the model snake test reflect active and avoidant behaviors separately, the single factor underlying anxiety-like behavior in the human intruder test consists of both active and avoidant behaviors. A potential explanation for this differential pattern of behavior between high and low imminence threat (Figure 4) is that high imminence threat necessitates the selection of a specific strategy e.g., active coping vs. avoidance. Evolutionarily, being able to choose between one or other of these two different coping behaviors may allow for higher chances of survival overall, dependent on whether the predator is deterred by active engagement (predators that rely on remaining unnoticed, e.g., snakes) or is not deterred, and therefore the more appropriate course of action would be to avoid and flee (Crofoot, 2012). In contrast, when the threat has low imminence, selecting one option over another is not immediately required and thus the animals show a combination of behaviors more consistent with threat appraisal and risk assessment (Blanchard et al., 2011).

When comparing animal scores on the two factors of the model snake test, it was evident that there was marked individual variation. Noticeably, most animals either showed high scores on the avoidant coping factor but low scores on the active coping factor (avoidant copers), or high scores on the active coping factor but low scores on the avoidant coping factor (active copers). The other animals either had high scores on both the active and avoidant coping factors (mixed copers) or showed low scores on both factors and thus appeared relatively unreactive to the snake overall (low coping behavior). Since 76% were either active or avoidant copers, this suggests that most animals tend to have a predominant coping style. This is consistent with the finding that although most people use both active and avoidant coping strategies in response to stressful situations, individuals tend towards a bias in using one type over the other, reflecting their coping styles (Folkman and Lazarus, 1980).

The finding that avoidant copers in response to the snake showed the greatest responsivity to the ambiguous situation created by the human intruder is consistent with human studies with self-reported measures, and the high comorbidity of avoidant personality disorder and anxiety disorders (Skodol et al., 1995). Furthermore, individuals who changed from an active to avoidant coping style showed an increase in depressive symptoms, whereas individuals who did the opposite showed a decrease (Herman-Stabl et al., 1995). It is important to note that mixed copers which not only showed high avoidant coping but also highly active coping in response to the snake displayed lower anxiety-like behaviors in response to low imminence threat, similar to active copers, compared to avoidant copers. This highlights the fact that high anxiety-like behavior in the face of uncertainty and ambiguity was linked specifically to the combination of both high avoidant and low active coping when the threat was highly imminent.

Adopting an avoidant coping style can reduce stress acutely by the removal of the individual from the stress-provoking environment, but may lead to prolonged stress in the future as the source of the stress is not overcome (Bardeen, 2015). In addition, natural processes that serve to reduce anxiety and conditioned threat responses such as desensitization or fear extinction are not experienced if the threat is wholly avoided. Consequently, the individual’s avoidant coping style is reinforced *via* negative reinforcement and may gain predominance over active coping impulses. Taken together, a predominantly avoidant behavioral pattern to high imminence threat and a lack of active coping behaviors may be maladaptive as it impedes the resolution of threat *via* threat engagement/confrontation, leading to an increased vulnerability to anxiety.

In summary, factors characterizing behaviors in response to low imminence and high imminence threat were identified in the common marmoset on the human intruder and model snake tests respectively. Our findings of distinct factors representing avoidant and active coping support the bimodal theory of defensive behavior under high stress and threatening contexts. Taking an analytical approach to modeling the full repertoire of an animal’s behavior revealed a differential pattern of active and avoidant behavioral responses as threat imminence is appraised.

With the factors identified, we demonstrate that a primarily avoidant coping style is associated with higher levels of anxiety-like behavior in response to low imminence threat, implicating a link between a predominantly avoidant coping strategy under high imminence threat and heightened sensitivity to uncertain/ambiguous situations associated with low imminence threat. These findings emphasize the importance of active coping strategies to alleviate stress reactivity and in helping individuals with avoidant coping styles suffering from excessive anxiety and fear. Insights from the paradigms described here promise to guide subsequent work interrogating the brain circuits involved in the control of active and avoidant coping behaviors and should facilitate translation to humans.

## Data Availability Statement

The datasets generated for this study are available on the Cambridge research repository Apollo (doi: 10.17863/CAM.49881) and available on request to the corresponding author.

## Ethics Statement

The animal study was reviewed and approved by an Ethical Review Committee from the University of Cambridge and conducted in accordance with the project and personal licenses held by the authors under the UK Animals (Scientific Procedures) Act of 1986.

## Author Contributions

SQ conducted all analyses and prepared the manuscript. AS and AR contributed to the data and results analyses and to the manuscript, as joint senior authors. GC and LM performed behavioral testing and scoring.

## Conflict of Interest

The authors declare that the research was conducted in the absence of any commercial or financial relationships that could be construed as a potential conflict of interest.

## Footnotes

^1^http://www.jwatcher.ucla.edu/
