# Supplementary material for: Avoidant Coping Style to High Imminence Threat Is Linked to Higher Anxiety-Like Behavior
Source: Front Behav Neurosci. 2020 Mar 10;14:34. doi: 10.3389/fnbeh.2020.00034 (PMC7078632; doi:10.3389/fnbeh.2020.00034)
Supplement: Supplementary file 1 [file Data_Sheet_1.PDF]

Supplementary Materials

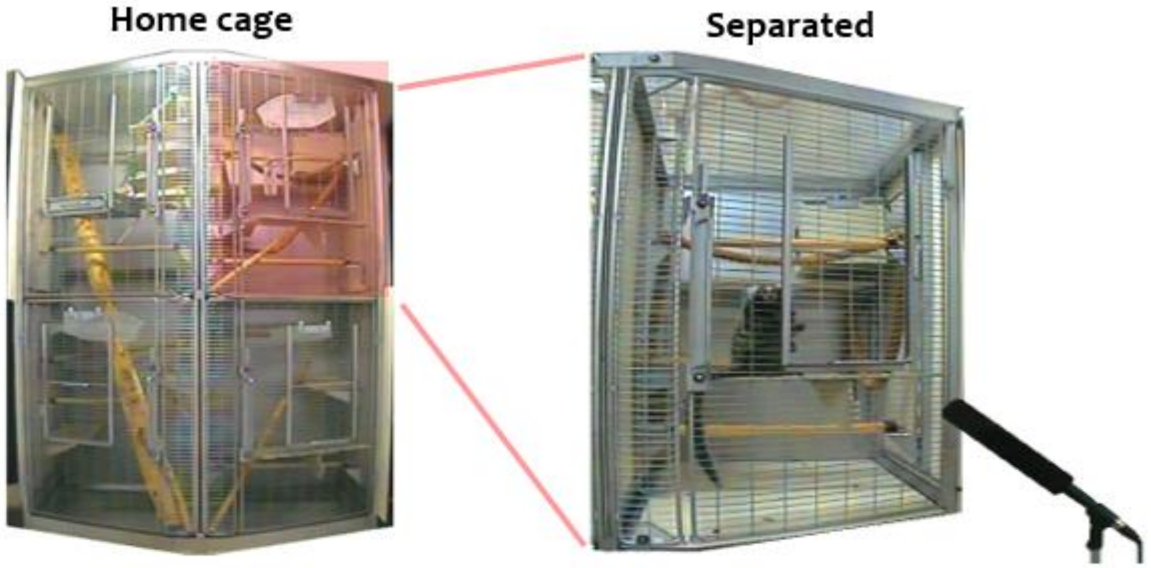

Supplementary Figure 1: Human intruder and model snake test setup. Animals are separated from their cagemate within the top-right quadrant of their homecage (highlighted in pink) with solid opaque dividers for the duration of the human intruder and model snake test. The angling of the left and right sides of the cage ensures that the test subject and cagemate do not have visual contact during the test.

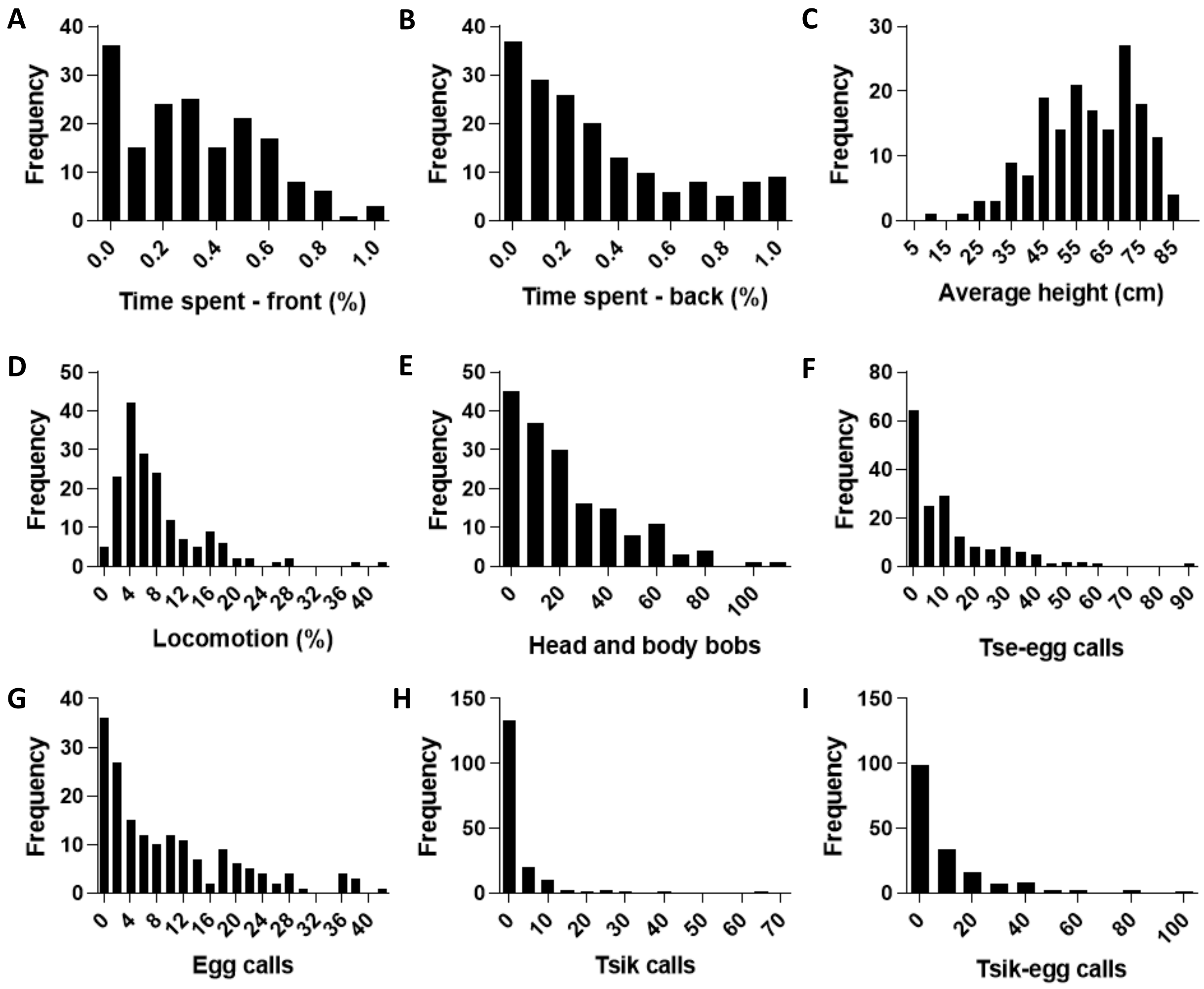

Supplementary Figure 2: Frequency histograms of human intruder test behavioral measures used in the EFA. (**A**) time spent at front, (**B**) time spent at back, (**C**) average height, (**D**) locomotion, (**E**) head and body bobs, (**F**) tse-egg calls, (**G**) egg calls, (**H**) tsik calls, and (**I**) tsik-egg calls. All variables display non-normal distribution.

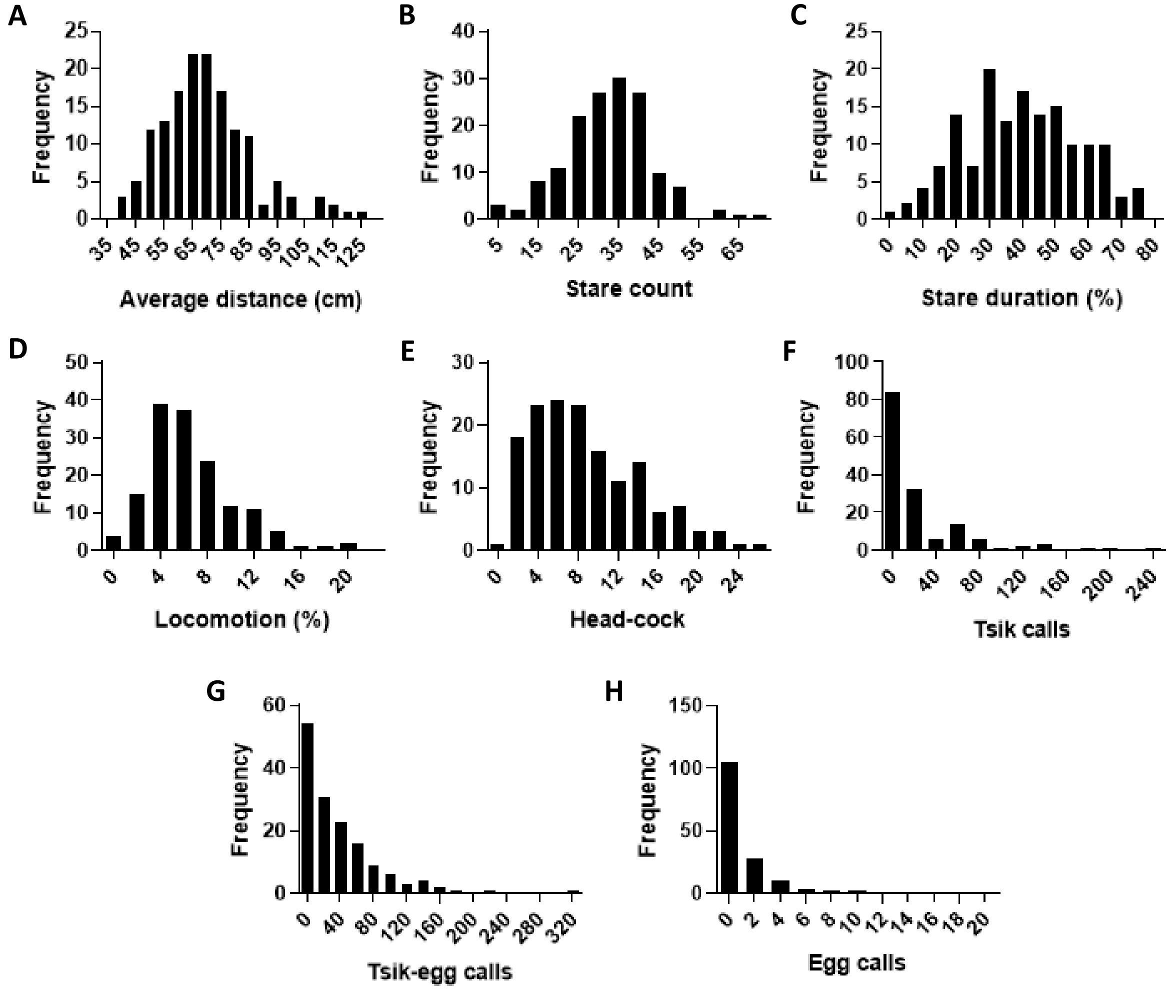

Supplementary Figure 3: Frequency histograms of rubber snake test behavioral measures in the EFA. (**A**) average distance, (**B**) stare count, (**C**) stare duration, (**D**) locomotion, (**E**) head-cock, (**F**) tsik calls, (**G**) tsik-egg calls, and (**H**) egg calls. All variables display substantial non-normal distribution.

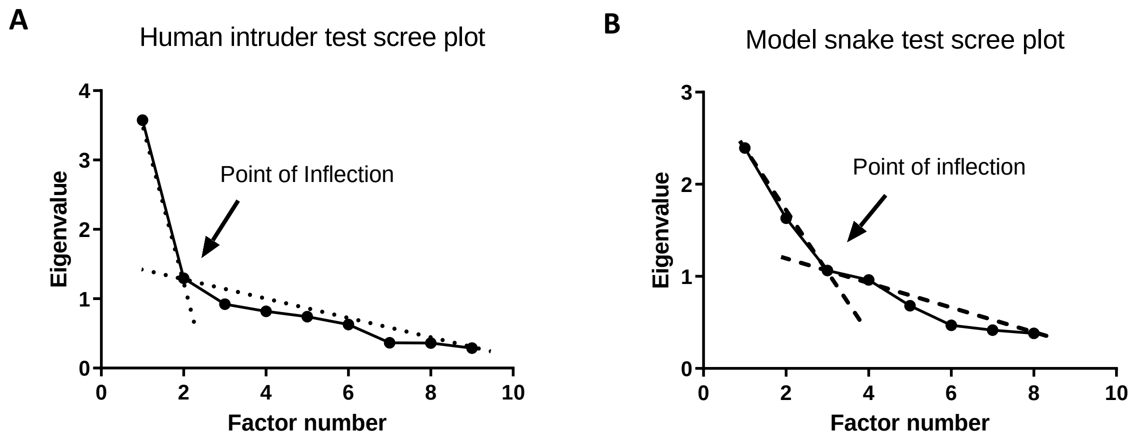

Supplementary Figure 4: Scree plots for the human intruder test and the model snake test. The point of inflection suggests (**A**) 1 factor should be extracted from the EFA of the human intruder test and (**B**) 2 factors should be extracted for the model snake test.

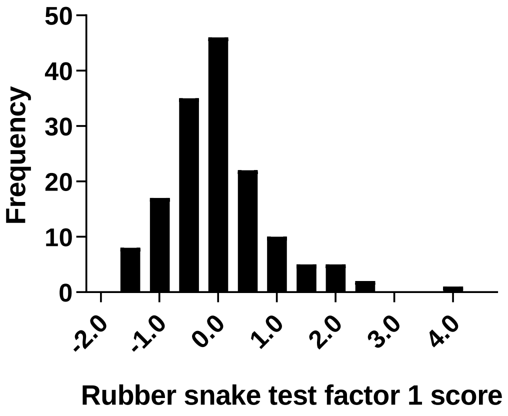

Supplementary Figure 5: Frequency histograms of the factor score representing active coping in the rubber snake test. The factor score distribution reflect significantly non-normal skewness and kurtosis.
